# Cytomegalovirus-associated splenic infarcts in an adult immune-competent man: a case report and review of the literature

**DOI:** 10.1186/1752-1947-8-85

**Published:** 2014-03-04

**Authors:** Μaria Νikoletta Protopapa, Dimitrios Velissaris, Athena Mougiou, Dimitrios Siagkris

**Affiliations:** 1 Department of Internal Medicine, University Hospital of Patras, Rio Patron, 26500 Patras, Greece; 2 Department of Haematology, University Hospital of Patras, Rio Patron, 26500 Patras, Greece

**Keywords:** Cytomegalovirus, Immune-compromised host, Hepatosplenomegaly, Splenic infarct

## Abstract

**Introduction:**

Cytomegalovirus-associated thrombosis has been extensively reported in the medical literature, mainly in immune-compromised patients. However, the association with splenic infarcts has been rarely mentioned.

**Case presentation:**

We report the case of a 38-year-old Caucasian man of Hellenic origin with acute cytomegalovirus infection presenting with spontaneous splenic infarcts. Echocardiography did not show any vegetations or mural thrombi. Anticoagulation treatment was not considered due to implication of minor vessels and since cytomegalovirus was the probable trigger for thrombosis in this patient.

**Conclusions:**

This case report serves as additional evidence for the role of cytomegalovirus in thrombosis.

## Introduction

Acute infection with cytomegalovirus (CMV) is usually asymptomatic or may present as an infectious mononucleosis like-syndrome. In the immune-compromised patient, however, the clinical setting of the disease can be expressed with various life-threatening conditions. CMV-associated thrombosis has been extensively reported in the medical literature, mainly in immune-compromised patients. However, the association with splenic infarcts has been rarely mentioned [1,2]. While most reports of CMV-associated thrombosis are referring to immune-compromised transplant recipients or human immunodeficiency virus (HIV)-positive patients, we report a case of acute CMV infection in an immune-competent patient, presenting with spontaneous spleen infarcts.

## Case presentation

A previously healthy, 38-year-old Caucasian obese (body mass index (BMI) 32) man of Hellenic origin, presented with a three-week history of hyperpigmented urine, a two week history of malaise and weakness and a 10-day history of high-grade fever associated with a nonproductive cough and no obvious weight loss. During this period, he had been documenting daily temperatures of 37°C to 39°C. On admission, our patient was not receiving any medication and his medical history was irrelevant. He had not been in recent contact with animals or ill people, was only a social drinker and not a regular user of tobacco products or drugs. As a professional driver, he had travelled regularly in the preceding months.

A physical examination on admission showed an axillary temperature of 38°C, a pulse rate of 110 beats per minute (bpm) and a normal blood pressure. Cardiac auscultation revealed regular S1, S2 clearly perceived with no associated murmurs. Lung auscultation was unremarkable and an abdominal examination revealed hepatomegaly and splenomegaly palpable to an extension of 3cm beneath his left rib cage. There was no lymphadenopathy or signs of meningismus. There had been apparent lower limb edema and no associated tonsillar findings.

His laboratory test results demonstrated a white blood cell count of 13,660/μL with 63.8 percent lymphocytes. Activated lymphocytes were observed on blood smear. Alanine transaminase and aspartate transaminase levels were mildly elevated (119 and 67U/L, respectively), while gamma-glutamyltranspeptidase (γGT), alkaline phosphatase (ALP) and lactate dehydrogenase (LDH) were also high.

Our patient’s laboratory test results on admission, on discharge and at one month follow-up are described in Table 1.

**Table 1:** Laboratory data pre-/post-admission

**Laboratory results on admission**			
**General**			
	**Admission**	**Discharge**	**1 Month later**
**WBC**	**13660/μl (range 4000-11000/μl)**	**8780**	**7700**
**NEUT**	**19.2% (range 50-70%)**	**33.6**	**42.4**
**LYMPH**	**63.8% (20-40%)**	**56.3**	**48.1**
**MONO**	**11.6% (range 0-8%)**	**8.9**	**8.2**
**Hct**	**38% (range 36–52%)**	**37.6**	**40.3**
**Hgb**	**13.3gr/dl (11.8-17gr/dl)**	**12.6**	**13.3**
**PLTs**	**176,000/μl (range 150,000-400,000/μl)**	**279,000**	**330,000**
**MCV**	**85fl (range 79-98fl)**	**87,2**	**86,5**
**ESR**	**20 (range 0–10)**	**10**	**8**
**CRP**	**6.7U/L (range <0.5U/L)**	**3**	**1.36**
**Microscopic appearance**			
**Activated lymphocytes**	**Many**		
**Coagulation profile – thrombophilia screening**			
	**Admission**	**Discharge**	**1 Month Later**
**PT**	**13.8sec (ref 13)**	**14**	**13,6**
**aPTT**	**35.6sec (ref 24–36)**	**37.7**	**34.2**
**INR**	**1.08 (ref 1)**	**1.10**	**1.06**
**D-dimers**	**3,93μg/ml (ref 0–0,5)**	**5.13**	**2.24**
**Fibrinogen**	**338mg/ml (ref 200–400)**	**369**	**479**
**Homocysteine**		**8.47μmol (ref 6.3-11.2)**	**Normal**
**APA**	**Negative**		
**ACA**	**Negative**		
**LA**	**Negative**		
**Anti-thrombin III**	**88% range 80-120**		
**Protein S**	**52% (normal values 65–140)**	**71%**	
**APC-R**	**153sec (normal >120)**		
**Protein C**	**74% (normal range 70–130)**	**75%**	
**FV-Q506 (Leiden)**	**Not detectable**		
**FII (G20210A)**	**Not detectable**		
**MTHFR (C677T)**	**Heterozygote**		
**Anti-β2-glycoprotein I (β2GPI-I)**	**Negative**		
**FV (H1299R)**	**Not detectable**		
**MTHFR (A1298C)**	**Not detectable**		
**Serologic tests**			
	**Discharge**	**1 Month later**	
**EBV**	**Negative**		
**HBV**	**Negative**		
**HCV**	**Negative**		
**HIV**	**Negative**		
**CMV IgM**	**Positive**		
**CMV PP65**	**Positive**		
**ANA**	**Negative**		
**ANCA**	**Negative**		
**C3**	**158mg% (range 79.0-152.0)**		
**C4**	**21.3mg% (range 16.0-38.0)**		
**PNH**	**Negative**		
**Biochemical profile**			
	**Admission**	**Discharge**	**1 Month Later**
**Ca**	**8,6 mg/dl (range 8,8-10,4)**	**9,1**	**9,5**
**Mg**	**2,2 mg/dl (range 1,6-2,3)**	**2**	**2,2**
**Glu**	**110 mg/dl (range 75-115)**	**86**	**91**
**Urea**	**26 mg/dl (range 15-54)**	**27**	**30**
**Crea**	**1 mg/dl (range 0,9-1,6)**	**1**	**1**
**Ur acid**	**4,1 mg/dl**	**4**	**4**
**TP**	**6,3 mg/dl (range 6-8,4)**	**6,5**	**6,4**
**Alb**	**3,3 mg/dl (range 3,5-5,5)**	**3,2**	**3,4**
**Glob**	**3 mg/dl (range 2,5-3,4)**	**3,3**	**3**
**Bil tot**	**1,07 mg/dl (range 0,1-1,3)**	**0,96**	**1,14**
**SGOT**	**67 U/L (range 5-40)**	**41**	**28**
**SGPT**	**119 U/L (range 5-40)**	**93**	**57**
**γGT**	**294 U/L (range 10-50)**	**296**	**200**
**LDH**	**382 U/L (range 120-230)**	**339**	**219**
**CPK**	**64 U/L (range < 190)**	**47**	**59**
**ALP**	**182 U/L (range 34-104)**	**175**	**139**

WBC, white blood cells; Neut, neutrophils; Lymph, lymphocytes; Mono, monocytes; Hct, hematocrit; Hgb, hemoglobin; PLTs, platelets; MCV, mean cell volume; ESR, erythrocyte sedimentation rate; CRP, C-reactive protein; INR, international normalized ratio; aPTT, activated partial thromboplastin time; PT, prothrombin time; DD, D-dimers; APA, antiphospholipid antibodies; ACA, anticardiolipin antibodies; LA, lupus anticoagulant; APCR, activated protein C resistance; FV, factor V; FII, factor II; MTHFR, methylene-tetra-hydro-folate reductase; AFV, activated factor V; AFVIII, activated factor VIII; ALP, alkaline phosphatase; BMI, body mass index; CMV, cytomegalovirus; DVT, deep venous thromboembolism; EBV, Epstein-Barr virus; HBV, hepatitis b virus; HCV, hepatitis c virus; HIV, human immunodeficiency virus; ANA, anti-nuclear antibodies; ANCA, anti-neutrophilic cytoplasmic antibodies; C3, complement 3; C4, complement 4; PNH, paroxysmal nocturnal haemoblobinuria; PP65, phosphoprotein 65; Ca, calcium; Mg, magnesium; Glu, glucose; Crea, creatinine; Ur, uric acid; Tp, total proteins; Alb, albumin; Glob, globulins; Bil tot, total bilirubin; SGOT, serum glutamic oxaloacetic transaminase; SGPT, serum glutamic pyruvic transaminase; γGT, gamma-glutamyltranspeptidase; LDH, lactate dehydrogenase; CPK, creatinine phospho-kinase; ALP, alkaline phosphatase.

**Figure 1:**
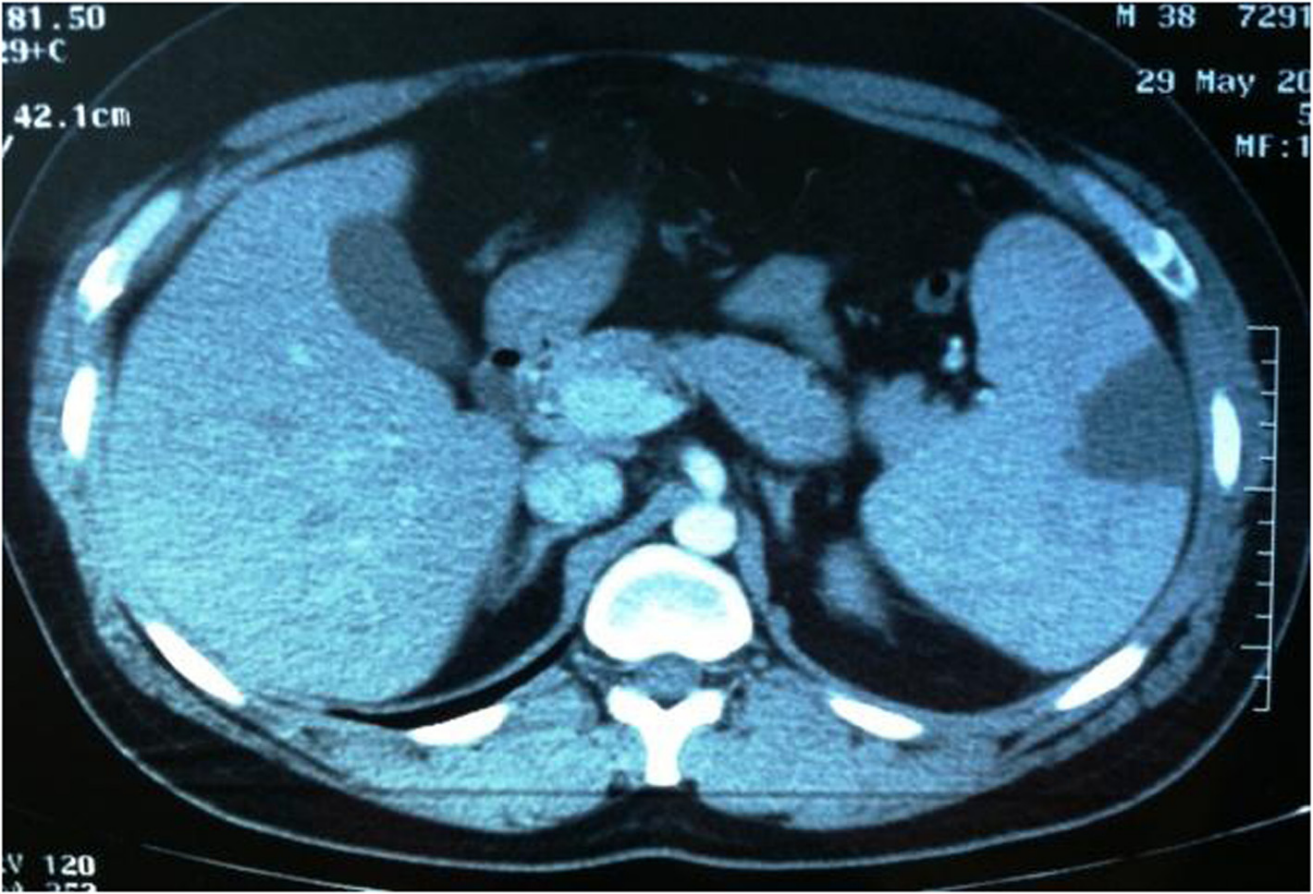
Splenomegaly associated with abnormal low-density sphenoid-type mass located at the outer left part of the organ.

An abdominal ultrasound scan revealed hepatosplenomegaly with a raised suspicion for a splenic infarct. A computed tomographic angiogram (CTA) was performed and confirmed the presence of the infarct as well as the hepatosplenomegaly without any other occlusive findings. Neither splenic artery nor vein thrombosis was identified (see Figure 1).

His blood and urine cultures were sterile and transesophageal echocardiography did not show any vegetations or mural alterations. His serologic test results for Epstein-Barr virus (EBV), hepatitis B, hepatitis C and HIV were negative, while his serologic test results for CMV immunoglobulin M (IgM) antibodies were positive. A positive CMV phosphoprotein 65 (PP65) antigenemia assay confirmed the diagnosis of acute CMV infection, but a prior hypercoagulable state was still considered. Prothrombin time (PT) value (15.5 seconds reference time 13), international normalized ratio (INR) value (1.25 reference approximately 1), activated partial thromboplastin time (aPTT) value (43.6 seconds range 24 to 36) and D-dimers (DD) levels (5.20μg/ml range 0 to 0.5) were elevated, fibrinogen (391mg/dl range 200 to 400) and homocysteine (8.47μmol/L range for men 30 to 59 years 6.3 to 11.2) were within normal range. His antiphospholipid antibodies (APA) panel and anticardiolipin antibodies (ACA) were negative. Finally, the rest of the exams were as follows: antithrombin 88 percent (range 80 to 120), protein C 74 percent (range 70 to 130) and protein S 52 percent (range 65 to 140). Factor V-Q506 (Leiden) and FII (G20210A) were negative.

At this point, anticoagulation treatment was considered, but it was ruled out since CMV was the probable trigger or cause of the thrombosis in our patient and as the majorly implicated vessels were minor ones. Ganciclovir treatment was also considered but not administrated since it is an agent primarily indicated for immune-compromised and occasionally in immune-competent patients with target-organ involvement.

An abdominal ultrasound scan four days after admission revealed no significant changes and our patient was subsequently discharged from hospital two days after without being given any medication, with the diagnosis of CMV infection with associated splenic infarct. Our patient was advised to follow a regular check on an outpatient basis in a months’ time with ultrasound control and monitoring of the relevant blood parameters. He was also encouraged to undergo a control blood sampling one month after and the examination revealed a substantial increase in protein S levels and a normalization of all other biochemical parameters. The ultrasound check performed the same day confirmed that the splenic echogenicity return to normal.

## Discussion

We report the case of an immune-competent man with concomitant acute CMV infection and thrombosis, an association reported previously in the medical literature mainly in immune-compromised patients. The exact pathologic mechanism by which CMV triggers thrombosis is still unclear. Current theories suggest that CMV induces thrombosis by promoting platelet and leukocyte adhesion to infected endothelial cells, or, alternatively, by increasing the circulatory levels of factor VIII. Other theories suggest that CMV induces transient APA production and enhances vascular smooth-muscle proliferation. Genetic predisposing factors for thrombosis in patients with CMV-associated thrombosis, such as factor V Leiden mutation, were also previously reported [3]. CMV infection is also known to lead to elevation of both platelet-derived growth factor and transforming growth factor-β. These growth factors can cause vascular cell wall proliferation [4]. It has been shown that CMV infection can also cause vascular cell activation and expression of adhesion proteins leading to increased platelet and leukocyte adhesion. This proinflammatory effect can cause changes that alter the anticoagulant environment of the vascular endothelium so that it favors coagulation [5]. CMV has also been shown to increase the levels of interleukin (IL)-1β, IL-6, tumor necrosis factor (TNF)-α, and other cytokines that have inflammatory properties [6,7]. Finally, few cases of immunologic alterations, such as cryoglobulinemia have been described in the acute phase of primary CMV infection in immune-competent patients [8]. Therefore, CMV infection, as a result of either one or a combination of these mechanisms, can induce vascular changes that may trigger a cascade of events that lead to inflammation and thrombosis [9]. Regardless of what the causative mechanism is, there is growing evidence that CMV infection may induce vascular damage with associated thrombosis that may be life-threatening.

Our initial hypothesis has been that of a suppressive impact of CMV upon the mechanism of action of protein S (PS) rather than that of an eventual mechanical endothelial damage. As is well known, PS is a vitamin K-dependent anticoagulant protein. It mainly functions as a cofactor to facilitate the action of activated protein C (APC) on its substrates, activated factor V (FVa) and activated factor VIII (FVIIIa). To support the significance of a low PS level we should co-evaluate the existence of a laboratory proof of activated protein C resistance (APCR). APCR is the inability of protein C to cleave FVa and/or FVIIIa, which allows for longer generation of thrombin and may lead to a hypercoagulable state. Protein S deficiency, on the other hand, may be hereditary or acquired, the latter being usually due to hepatic diseases, nephrotic syndrome, a vitamin K deficiency or autoantibodies. Autoantibodies to PS may form immune complexes, inducing increased clearance of PS or interfering with the protein C-protein S system [10]. Protein S deficiency usually manifests clinically as venous thromboembolism (VTE). The association of PS deficiency with arterial thrombosis appears less and weaker. Arterial thrombosis is not evident with other hereditary anticoagulant abnormalities (for example PC or antithrombin deficiency, and factor V Leiden gene mutation). The normalization of PS concomitant to the cessation of the clinical manifestations of the CMV infection appears to be substantial to the sustainment of our hypothesis, although protein S is usually consumed during the acute phase of thrombosis to counterbalance the prothrombotic factors so the rise of PS a month after could also be explained by this phenomenon. Therefore, there is not really strong evidence in favor of PS as major a mechanism of CMV thrombosis. Other concomitant risk factors inevitably contribute to thrombosis provocation.

Fever accompanied by splenic infarcts in an immune-competent patient can be seen in endocarditis, in viral infections such as EBV, in infectious vasculitis as observed in neisserial infections and in various other noninfectious conditions, including sickle cell anemia, autoimmune vasculitis and hypercoagulable states. Extensive reports on concomitant acute CMV infection and thrombosis in immune-competent patients exist [1,2,11,12]. Moreover, reports on acute CMV infection and splenic infarcts in immune-competent patients are even more rare. In the immune-competent host with CMV, vasculitis with thrombosis appears to be extremely rare. We identified very few cases reported in the literature [2,13-16]. Two 31-year-old women with apparently active CMV disease developed hepatic vein thrombosis but both patients were taking oral contraceptive pills, which predispose patients to thrombosis [17,18]. A four-month-old girl with acute CMV infection also developed portal vein thrombosis, but she had PC and PS deficiency, which very likely contributed to thrombosis in this patient [19]. A 30-year old woman with a two-week history of fever, myalgia and cough showed a transiently elevated anticardiolipin IgG with a normal anticardiolipin IgG and a negative lupus anticoagulant [13]. A meta-analysis showed that deep venous thromboembolism/pulmonary embolism (DVT/PE), splanchnic vein thrombosis and splenic infarction were the most prevalent thromboses associated with acute CMV infection with splanchnic vein thrombosis being the one most prevalent among immune-competent patients [2]. Clinical and laboratory observations in nine other cases, in other studies, demonstrated cytomegalovirus mononucleosis to be common among young adults giving credit to the fact that this diagnosis should be considered in all patients with detected mononucleosis-like antibodies, including those with lymphadenopathy and splenomegaly [16].

## Conclusions

CMV is a rare, but potentially significant, cause or precipitating factor for thrombosis in immune-competent hosts. We think that all patients with unexplained fever and spontaneous thrombosis should be screened for CMV infection; in cases of splenic infarcts due to thromboembolism or congenital hypercoagulability, anticoagulation is mandatory, while in cases of splenic infarcts alone, CMV infection may influence the decision on whether or not to start anticoagulation therapy. Many theories on the mechanism of interaction between CMV and human organisms have been postulated, a fact that makes evident the need for further investigation and clinical observation.

This case report serves as additional evidence for the role of CMV in thrombosis. Cytomegalovirus-associated vascular thrombosis is a rare but increasingly reported phenomenon and should be considered in the differential diagnosis of splenic infarction once more common diagnoses, such as endocarditis and lymphoma, have been excluded.

## Consent

Written informed consent was obtained from the patient for publication of this case report and any accompanying images. A copy of the written consent is available for review by the Editor-in-Chief of this journal.

## Abbreviations

WBC: white blood cells; Neut: neutrophils; Lymph: lymphocytes; Mono: monocytes; Hct: hematocrit; Hgb: hemoglobin; PLTs: platelets; MCV: mean cell volume; ESR: erythrocyte sedimentation rate; CRP: C-reactive protein; INR: international normalized ratio; aPTT: activated partial thromboplastin time; PT: prothrombin time; DD: D-dimers; APA: antiphospholipid antibodies; ACA: anticardiolipin antibodies; LA: lupus anticoagulant; APCR: activated protein C resistance; FV: factor V; FII: factor II; MTHFR: methylene-tetra-hydro-folate reductase; AFV: activated factor V; AFVIII: activated factor VIII; ALP: alkaline phosphatase; BMI: body mass index; CMV: cytomegalovirus; DVT: deep venous thromboembolism; EBV: Epstein-Barr virus; HBV: hepatitis b virus; HCV: hepatitis c virus; HIV: human immunodeficiency virus; ANA: anti-nuclear antibodies; ANCA: anti-neutrophilic cytoplasmic antibodies; C3: complement 3; C4: complement 4; PNH: paroxysmal nocturnal haemoblobinuria; IL: interleukin; IgM: immunoglobulin M; PC: protein C; PE: pulmonary embolism; PP65: phosphoprotein 65; PS: protein S; TNF: tumor necrosis factor; Ca: calcium; Mg: magnesium; Glu: glucose; Crea: creatinine; Ur: uric acid; Tp: total proteins; Alb: albumin; Glob: globulins; Bil tot: total bilirubin; SGOT: serum glutamic oxaloacetic transaminase; SGPT: serum glutamic pyruvic transaminase; γGT: gamma-glutamyltranspeptidase; LDH: lactate dehydrogenase; CPK: creatinine phospho-kinase; ALP: alkaline phosphatase.

## Competing interests

The authors declare that they have no competing interests.

## Authors’ contributions

PMN made substantial contribution to the conception, acquisition and interpretation of data. VD contributed to the analysis of data and the primary revision of manuscript. MA contributed to reviewing the manuscript and giving final approval of the version to be published. SD has been involved in critically revising the manuscript for important intellectual content and giving final approval to the manuscript. All authors read and approved the final manuscript.
